# Postoperative Monitoring After Elective Intracranial Surgery in a Postanesthesia Care Unit is Safe, Efficient, and Cost-Effective

**DOI:** 10.1007/s12028-025-02323-z

**Published:** 2025-07-21

**Authors:** Arthur Wagner, Alexander Quiring, Anna Do, Markus Heim, Gerhard Schneider, Bernhard Meyer

**Affiliations:** 1 Department of Neurosurgery, University Hospital, Technical University Munich School of Medicine, Munich, Germany; 2 Department of Anesthesiology, University Hospital, Technical University Munich School of Medicine, Munich, Germany

**Keywords:** Postanesthesia care unit, Intensive care unit, Postoperative monitoring, Resource use, Complication management

## Abstract

**Background:**

This study evaluates whether monitoring patients in the postanesthesia care unit (PACU) after elective intracranial surgery is as safe and effective as intensive care unit (ICU) monitoring, focusing on postoperative complications and resource use.

**Methods:**

A retrospective cohort study was conducted at a tertiary academic hospital, analyzing patients who underwent elective craniotomies from March 2013 to September 2023. Patients were allocated to PACU or ICU monitoring based on preoperative risk assessment and intraoperative events. Outcomes measured included complication rates, revision surgeries within 72 h, transfers from PACU to ICU, and ICU resource use.

**Results:**

A total of 5638 consecutive patients (mean age 54 years, 56% female) were analyzed, of whom 96.0% were monitored in the PACU, whereas 3.7% required ICU admission due to high-risk conditions or intraoperative events. The early complication rate in PACU-monitored patients was 17.1%, with most complications occurring within the first 13.2 h (SD 17.0) post surgery. Revision surgery was required in 4.6% of cases, and transfers from the PACU to the ICU occurred in 1.9%. PACU monitoring reduced ICU use by 94.4% compared to an all-ICU strategy, with no compromise in safety or outcomes.

**Conclusions:**

PACU-based monitoring is a safe and efficient alternative to ICU care for elective craniotomy patients, offering comparable complication rates and outcomes. This approach significantly reduces ICU demand, providing a cost-effective strategy that optimizes critical care resources while maintaining patient safety.

## Introduction

Postoperative monitoring following elective craniotomy for various pathologies is a crucial aspect of neurosurgical care because it carries unforeseen risks in a considerable number of patients, mainly within the first 24 h after surgery. These risks necessitate a rigorous and comprehensive postoperative monitoring strategy to ensure early detection and management of complications [1–3]. 

Historically, these patients were monitored in specialized intensive care units (ICUs), and they still are in most institutions. However, the ICU is resource intensive, and its limited availability can pose challenges, especially in high-volume centers [4, 5].

In recent years, the postanesthesia care unit (PACU) has emerged as a viable alternative for postoperative monitoring. The PACU provides a dedicated environment for immediate postoperative care, with capabilities for intensive monitoring and management similar to the ICU. Implementing PACU-based monitoring strategies aims to optimize resource use without compromising patient safety [6].

Our departments initiated a recovery room surveillance concept for these patients as early as 2006, officially implemented the concept described herein more than a decade ago, and recently named it officially a PACU concept. Our study aims to evaluate the safety and efficiency of PACU-based monitoring in patients undergoing elective craniotomy for various pathologies in a large cohort of patients.

## Methods

### Study Design

This retrospective cohort study was conducted at the Departments of Neurosurgery and Anesthesiology of a major academic tertiary referral center. The study included all consecutive patients who underwent elective craniotomy for the resection or open biopsy of intracranial neoplasms as well as treatment of vascular lesions. The strategies for postoperative patient monitoring described herein were established in 2006, whereas the study population analyzed was included from 2013, when hospital records were fully digitalized and completely available.

### Postoperative Management

Postoperatively, adult patients of at least 17 years of age undergoing elective craniotomies were generally admitted to an eight-bed PACU equipped with ventilation machines and the capacity to monitor vital parameters, including invasive blood pressure (IBP). It was staffed with advanced practice nurse anesthetists and anesthesiologists, who were instructed to monitor the patients regarding level of consciousness, neurological deterioration, and elevated pain levels according to the postoperative protocol of the operating neurosurgeon. During off-hours (late and night shifts, weekends), one dedicated anesthesiologist was available on call at the discretion of the PACU’s nursing staff.

Patients with external ventricular drainages were continuously monitored for intracranial pressure (ICP) levels and drainage volume. Laboratory workups were conducted regularly within the first 24 h after surgery (postoperative day 1 [POD1]) in the PACU, including a complete blood count and coagulation parameters, and otherwise, as needed, if abnormalities had been detected during the intraoperative course or substitutions of blood products were made. Both PACU and ICU staff were trained in neuroanesthetic care and performed necessary diagnostics such as computed tomography only after consultation with a neurosurgeon, as in a semiclosed model [7]. The patient population of the PACU was of miscellaneous specialties.

During morning on POD1, all PACU patients were visited by attending neurosurgeons, anesthesiologists, and nurses to triage their transit to the floor, an intermediate care unit (IMC), or the ICU. The PACU prepped patients requiring immediate revision surgery.

Selected patients were primarily admitted to the ICU if the preoperative anesthesiologic visit saw the patient in a very critical condition (American Society of Anesthesiologists [ASA] score ≥ 4) due to severe comorbidity or the patient was anticipated to need continued ventilation postoperatively. This was agreed upon in a consensus discussion between anesthesiologists and neurosurgeons for every individual case at least 24 h prior to surgery. Additionally, patients with some forms of multidrug-resistant bacteria were primarily transferred to isolation rooms in the ICU for routine postoperative monitoring, in keeping with internal hygiene regulations. Whenever an intraoperative adverse event was detected, the attending neurosurgeon and anesthesiologist conducted a joint clinical assessment and reached consensus on postoperative disposition; if the adverse event was judged sufficiently significant, the patient was admitted directly to the ICU rather than the PACU, ensuring an appropriate level of postoperative monitoring and support. The concept of a neurointensive care unit as a step-up unit with a strictly predefined protocol and according to individual patient-specific criteria for resource allocation had been established jointly between the Departments of Neurosurgery and Anesthesiology.

### Data Analysis

Data collection included patient demographics, preoperative ASA score, preoperative symptoms, the localization of the pathology, and the use of anticoagulants. The postoperative assessment focused on early postoperative complications within the first 72 h, revision surgery rates, and transferal pathways to and from the PACU, ICU, IMC, and regular ward. The time spent in the PACU until the transfer was recorded, as well as the timing of revision surgeries.

### Statistical Analysis

We used IBM SPSS in its 27th (ibm.com/products/spss-statistics) version for statistical analysis; the level of significance was defined a priori as *α* = 0.05. Descriptive statistics were used to summarize patient characteristics and complication rates, z-tests were used to determine significant differences between proportions, and *t*-tests were used for significant differences between means. A positive vote by the local institutional review board was obtained for study conduction beforehand (reference no. 5626-12). 

## Results

**Fig. 1:**
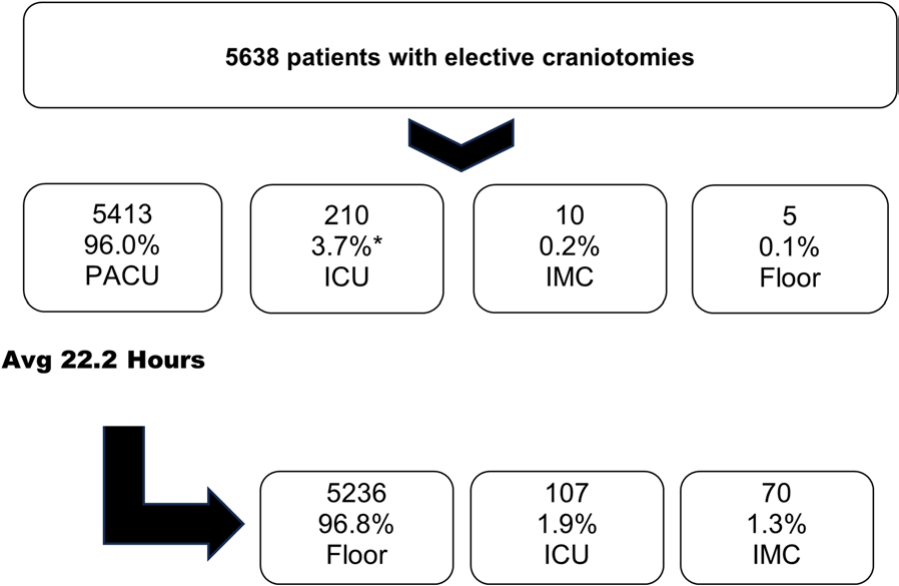
Flow diagram of postoperative patient pathways. Asterisk denotes this includes step-up allocation due to intraoperative adverse events. *Avg* Average, *ICU* Intensive care unit, *IMC* Intermediate care unit, *PACU* Postanesthesia care unit

Data from all 5638 patients who underwent elective craniotomy for various indications between March 2013 and September 2023 were extracted from digital hospital records. Baseline characteristics are listed in Table 1. Through an individual preoperative visit and consensus between the neurosurgical and anesthesiologic treating physicians, 98.5% (*n* = 5553) of the patients were scheduled for postoperative monitoring in the PACU, whereas 1.2% (*n* = 68) were primarily scheduled to the ICU due to critical conditions such as preexisting pulmonary and systemic infections (0.4%; *n* = 23), significant comorbidities (ASA score ≥ 4, 0.9%; *n* = 51; Table 2), and multidrug-resistant bacterial infections (0.1%; *n* = 6). In 2.5% (*n* = 141) of the originally PACU-allocated patients, intraoperative serious adverse events such as hemorrhage necessitated a conversion to the ICU (Fig. 1). Nine patients (0.3%) were monitored primarily in the IMC, and five patients (0.2%) were directly transferred to the regular ward, none of whom had to be stepped up to the ICU during our observation period. Patients who were secondarily transferred from the PACU to the ICU were generally older (mean age 69.8 vs. 57.2 years; *p* = 0.043) and more commonly had a higher ASA score (ASA score ≥ 4, 86.9% vs. 0.6%; *p* < 0.001; Table 2). Patients with intraventricular entities were more commonly transferred directly to the ICU (14.2% vs. 2.5%; *p* = 0.006; Table 2). Furthermore, we found that zero patients scheduled for elective intracranial surgery over the complete period had to be canceled due to the unavailability of postoperative monitoring.

**Table 1:** Baseline characteristics of cohort

	Value
Age at surgery, y	
Mean	54
Minimum	17
Maximum	82
SD	16
Female sex, *n* (% of total)	3152 (55.9)
Entity/surgery, *n* (% of total)	
Glioma	132 (28.9)
Meningioma	1024 (18.2)
Metastasis	971 (17.2)
Unruptured aneurysm clipping	734 (13.0)
Skull base repair	314 (5.6)
Cavernoma	155 (2.8)
Arteriovenous malformation	149 (2.6)
Microvascular decompression	108 (1.9)
Schwannoma	93 (1.7)
Pineocytoma/pineoblastoma	90 (1.6)
Other^a^	87 (1.5)
Ependymoma	61 (1.1)
Pituitary adenoma	56 (1.0)
Dural arteriovenous fistula	45 (0.8)
Lymphoma	29 (0.5)
Extracranial–intracranial bypasses	29 (0.5)
Hemangioblastoma	29 (0.5)
Craniopharyngioma	24 (0.4)
Medulloblastoma	8 (0.1)
Localization, *n* (% of total)	
Infratentorial	949 (16.8)
Supratentorial	4689 (83.2)
Blood thinner intake at time of surgery, *n* (% of total)	
None	4916 (87.2)
Antithrombotic	468 (8.3)
Anticoagulatory	242 (4.3)
Multiple	12 (0.2)
Preoperative symptoms, *n* (% of total)	
None	2047 (36.3)
Neurological deficit	2333 (41.4)
Seizure	194 (3.4)
Unspecific	1064 (18.9)

^a^“Other” includes neurocytoma, hemangioma, choroid plexus papilloma, neurofibroma, colloid cyst and epidermoid

**Table 2:** Subgroup analyses of variables between patient pathways

	Postoperative monitoring					
	PACU^a^	ICU^b^	PACU to ICU^c^	PACU to IMC^d^	Floor^e^	IMC^f^
Age in years, mean ± SD	57.2 ± 16.3	60.3 ± 16.9	69.8 ± 14.2^a^	58.8 ± 18.3	66.1 ± 7.4	52.4 ± 22.1
Female sex, *n* (%)	2975 (54.9)	94 (44.8)	32 (30.0)	40 (56.4)	5 (100.0)	6 (60.0)
Localization, *n* (%)						
Infratentorial	850 (16.0)	29 (15.8)	2 (10.0)	19 (18.8)	–	4 (23.5)
Supratentorial	4431 (81.5)^b^	135 (73.8)	18 (90.0)	80 (79.2)	3 (100.0)	11 (64.7)
Intraventricular	132 (2.5)	26 (14.2)^a,d^	–	2 (2.0)	–	2 (11.8)
Anticoagulation, *n* (%)						
None	4649 (87.6)	171 (93.4)^c^	15 (75.0)	88 (87.1)	3 (100.0)	15 (88.2)
Antithrombotic	430 (8.1)	10 (5.5)	3 (15.0)	4 (4.0)	–	–
Anticoagulatory	214 (4.0)	2 (1.1)	2 (10.0)	9 (8.9)	–	2 (11.8)^b^
Multiple	15 (0.3)	–	–	–	–	–
ASA, *n* (%)						
1	11 (0.2)	–	–	–	–	–
2	4480 (82.7)^b,c^	60 (28.6)	14 (13.1)	67 (95.7)^a,b,c,f^	5 (100.0)	8 (80.0)^b,c^
3	889 (16.4)^d^	99 (47.1)^a,d,f^	–	3 (4.3)	–	2 (20.0)^a,d^
4	33 (0.6)	51 (24.3)^a^	93 (86.9)^a,b^	–	–	–
5	–	–	–	–	–	–

Indices denote two-sided tests between categories with statistically significant differences at the *α* < 0.05 level

*ASA* American Society of Anesthesiologists score, *ICU* Intensive care unit, *IMC* Intermediate care unit, *PACU* Postanesthesia care unit

The PACU patients were monitored for an average of 22.2 h, with an SD of 17.5–26.5 h. Following this initial monitoring period, an additional 1.9% of patients (*n* = 106) were secondarily transferred to the ICU on POD1 (Fig. 1), primarily due to a prolonged decreased level of consciousness (1.1%; *n* = 61), agitation (0.5%; *n* = 28), and severe neurological disability (0.3%; *n* = 17). Thus, in a total of 94.4% of patients, ICU admission was avoided, saving 5321 ICU days in total.

**Fig. 2:**
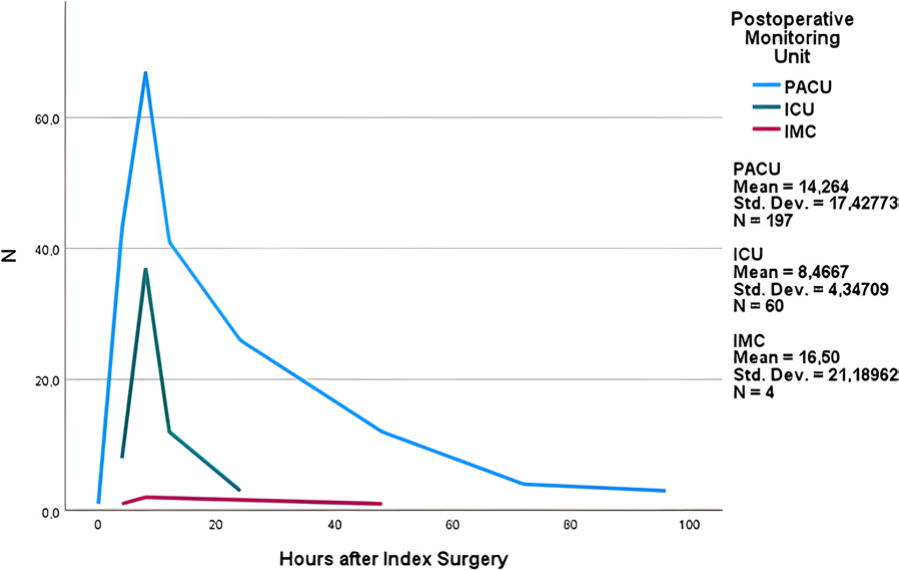
Histogram of revision surgeries or insertions of external ventricular drainages after index surgery, grouped by postoperative monitoring unit. *ICU* Intensive care unit, *IMC* Intermediate care unit, *PACU* Postanesthesia care unit

Overall, the early postoperative complication rate was 17.1% (*n* = 964), including new neurological deficit (12.3%; *n* = 694), rebleeding (6.5%; *n* = 361), and thromboembolism (4.3%; *n* = 239). An external ventricular drainage was inserted in 131 patients (2.3%). Most of these complications (*n* = 838; 82.8%) were detected while the patients were still under monitoring in the PACU. Significantly fewer complications were observed on POD2 (16.1%) and POD3 (5.1%; *p* = 0.038). Of the 261 cases (4.6%) in need of immediate revision surgery or implantation of external ventricular drainage, 92.0% (*n* = 240) occurred on POD1 during PACU monitoring, 5.0% (*n* = 13) occurred on POD2, and 3.0% (*n* = 8) occurred on POD3 (*p* < 0.001), with a mean of 13.2 h (median 8.0; SD 17.0; Fig. 2).

## Discussion

### The PACU in Historical Context

The concept of a PACU, as a structured recovery environment with extended monitoring capabilities and defined protocols, originated in the Anglo-American health care systems and began to emerge in our country during the 1990s. Initially, recovery rooms in hospitals functioned primarily as short-term postoperative holding areas, typically lacking continuous physician presence and advanced monitoring equipment. It was not until the early 2000s that a few larger academic centers—among which was ours—began implementing PACU-like structures to address ICU bed shortages and improve postoperative care pathways.

Despite growing acceptance, PACUs are still not legally or structurally defined as a separate unit within our health care system. Rather, they operate under the broader classification of “extended recovery rooms” and are subject to individual and institutional staffing regulations. The National Association for Intensive and Emergency Medicine and the National Society of Anesthesiology and Intensive Care Medicine have only more recently begun formalizing descriptions and standards for PACU functionality.

In this context, our institution introduced a recovery room monitoring concept as early as 2006 and established the infrastructure and triage protocols more than a decade ago, thus making it the first structured implementation of this model for elective neurosurgical procedures in our country, to the best of our knowledge. Our study offers a real-world evaluation of a mature PACU model within the unique legal and logistical framework of the national health care landscape.

### Efficiency of Monitoring in the Early Postoperative Phase

Neuro-ICUs are primarily designed and equipped to manage severe life-threatening diseases such as traumatic brain injury, intracranial hemorrhages, intracranial hypertension, or severe postoperative complications, as well as major systemic problems such as cardiac or respiratory failure [4, 8]. The vast majority of patients undergoing elective craniotomy do not fall into such categories. Thus, a valuable and expensive resource may be unavailable for either emergency or elective care, leading to cancelations and delays of elective craniotomies in the latter case [9]. Traditionally, neurosurgical patients are admitted to an ICU after surgery, only to be transferred to the ward on POD1 due to an uneventful postoperative course in most instances. Value-based care concepts contradict such an approach because it is neither efficient nor cost-effective and might even cause harm to patients.

Neurosurgeons and anesthesiologists at our hospital decided as early as 2006 to implement leaner PACU-like monitoring within the recovery rooms of the Operating Theatres by adapting them in a stepwise manner with, i.e., 24/7 staffing with concise human resources structures. The primary reason was to avoid cancelations of elective surgeries and the ever-present need for emergency beds in the ICU, jointly managed by neurosurgeons and anesthesiologists with equal rights. A genuine PACU concept was later developed, reducing direct and indirect costs by implementing key aspects of advanced monitoring facilities, i.e., ICP and IBP monitoring while cutting down on physician staffing [5, 6, 10]. PACUs also offer logistical advantages, as postoperative pathways are expedited after the transition from the PACU to a step-down unit. Azad et al. report a reduction in hospital length of stay of up to 4 days, which may factor into the overall reduction in expenses [4]. Similarly, our PACU concept had not been aimed at transferring ICU level equipment and staffing to a “transit ICU,” which becomes apparent in the differences of allocated facilities and resources between the units (see Table 3). A key difference in staffing compared to the ICU is the availability of a dedicated physician only on call during off-hours.

**Table 3:** Comparison of resource allocation between the neuro-ICU and PACU

	Neuro-ICU	PACU
Bed capacity	30	8
Nurse staff to bed ratio	1:2	1:4
Physician staff to bed ratio	1:2–3	1 (on call):8
Neurosurgical patients on average/24 h	14	3
Monitoring	ICP, IBP, ventilation parameters	ICP, IBP, ventilation parameters

*IBP* Invasive blood pressure, *ICP* Intracranial pressure, *ICU* Intensive care unit, *PACU* Postanesthesia care unit

### Allocation and Pathways of Monitoring

PACUs offer a dedicated environment for immediate postoperative care, with capabilities for monitoring and managing patients undergoing uneventful elective craniotomy similar to those in an ICU. As an aid in determining postoperative patient pathways, some institutions use predictive risk scores. However, most have failed to be validated accordingly and have yet to find their way into widespread adoption [2, 11–13].

Our subgroup analyses indicated significant differences in the characteristics of patients who required escalation from PACU to ICU, specifically in terms of age and ASA score. Patients transferred from the PACU to the ICU were significantly older compared to those who remained stable in the PACU and predominantly had higher preoperative ASA scores, with 86.9% classified as having an ASA score of 4, compared to just 0.6% of patients who stayed in the PACU without escalation. These findings are concordant with our preoperative assessment and actionable criteria, as outlined in the Methods section, in which a preoperative joint visit by anesthesiology and neurosurgery lays the basis for allocation to any postoperative pathway. It is thus only evident to stratify the preoperative assessment by the patients’ comorbidity load as mirrored in the ASA score, relegating patients with ASA scores of at least 4 to a direct ICU transfer. We would see the age criterion more differentiated, however. We believe that in the weighing of preoperative morbidity, the chronological age retains far less significance in comparison to the biological age as well as the overall functional capacity of the patient, and an excessive “scoring” of any and all functional dimensions may elude practicability in everyday practice. It seems prudent to primarily project the ASA score as a representative index for comorbidity—as has been common practice, not only at our institution—and relay chronological age to more of a surrogate and not an independent clear-cut criterion for ICU allocation. 

We decided early on a more pragmatic approach that identifies patients with preexisting severe comorbidity or foreseeable postoperative neurological issues in shared preoperative decision-making between surgeons and anesthesiologists. The necessity for intraoperative (2.5%) and immediate postoperative (1.9%) upgrades to higher care is low in our hands. These data show that our concept may be more feasible and valuable than scoring individual patients. In our experience, it facilitates a streamlined and uninterrupted workflow in which elective surgery cancelations and delays are exceptions. On the other hand, ICU capacities are free from fluctuations caused by the elective program and remain available to accept patients in critical conditions requiring intensive care [14–16]. 

### Safety of PACU Monitoring

Several studies have demonstrated that PACU-based monitoring does not compromise patient safety or quality of care. Young et al. reported on their “Safe Transitions Pathway,” which yielded significant cost savings [17]. Hoffman et al. report a 6-h reduction of time expedited until imaging when the patient was situated in the PACU, with similar 30-day readmission rates alongside a 7.4% cost reduction compared to a historical ICU cohort [6]. Another study by Florman et al. found that none of their 200 patients suffered from permanent neurological disability or mortality if managed by a step-down protocol from the PACU to the floor [18].

The safety and efficacy of PACU-based monitoring are supported by our complication rates, which are comparable to those reported in studies focusing on ICU-based postoperative care [19, 20]. This suggests that PACU-based monitoring does not compromise patient safety and can be a viable alternative to ICU care for most patients undergoing elective craniotomy. We found an early overall complication rate of 17.1%, necessitating revision surgeries and external drainage placements in 4.6%, which is comparable to rates reported in ICU-monitored populations [4, 15, 16, 18, 21]. The majority of these complications, including new motor deficits, rebleeding, facial nerve palsy, and new aphasia, occurred after a mean of 10 h. Similar to our results, studies report a peak complication rate within the first 6–24 h, which subsequently tapers off [22–24]. This stresses the approach of an early-accentuated monitoring protocol, as patients resided in the PACU during the peak incidence of complications, facilitating immediate intervention.

Although PACUs may not have the same level of resources as ICUs for managing severe systemic complications, they can stabilize patients and provide initial interventions before transferring them to the ICU if necessary. Our study found that only 1.9% of patients initially monitored in the PACU required an escalation of therapy and transition to the ICU, congruent with rates of 2.1–3.5% reported in the literature [4, 6, 18, 21]. The rationale for a secondary treatment escalation and transfer from the PACU to the ICU mainly concerns patients with a presumable continuing need for ventilation requiring intubation, typically for patients with significant intracranial masses and decreased levels of consciousness.

### Limitations and Strengths

This study has the limitation of being retrospective. However, the digital data of a consecutive cohort analyzed were complete and covered only a short perioperative period, in which data loss is usually minimal. On the other hand, the apparent strength lies in the large number of study participants included and the broad indication of elective craniotomies covered. It is more suitable as a real-life reference than most series published recently.

## Conclusions

Our study highlights the importance of streamlined patient pathways via a PACU to step-down unit fashion for postoperative surveillance of elective craniotomies instead of routine ICU admission. Individualized, interdisciplinary risk-based allocation proves highly accurate, obviating the need for scores. The same safety and efficacy as an all-ICU strategy can be achieved, ensuring consistent and high-quality patient care while preserving ICU resources and avoiding cancelations of elective surgeries.

## Data Availability

The data are available from the corresponding author upon reasonable request.
